# Detection and Phylogenetic Characterization of *Anaplasma capra*: An Emerging Pathogen in Sheep and Goats in China

**DOI:** 10.3389/fcimb.2018.00283

**Published:** 2018-08-30

**Authors:** Yongshuai Peng, Kunlun Wang, Shanshan Zhao, Yaqun Yan, Haiyan Wang, Jichun Jing, Fuchun Jian, Rongjun Wang, Longxian Zhang, Changshen Ning

**Affiliations:** ^1^College of Animal Science and Veterinary Medicine, Henan Agricultural University, Zhengzhou, China; ^2^International Joint Research Laboratory for Zoonotic Diseases of Henan, Zhengzhou, China; ^3^Experimental Research Center, Henan University of Animal Husbandry and Economy, Zhengzhou, China

**Keywords:** *Anaplasma*, *Anaplasma capra*, tick-borne diseases, sheep, goat, *gltA*, *msp4*, *groEL*

## Abstract

*Anaplasma capra* is an emerging pathogen, which can infect ruminants and humans. This study was conducted to determine the occurrence of *A. capra* in the blood samples of sheep and goats in China. Using nested polymerase chain reaction (nested-PCR) targeting the *gltA* gene and conventional PCR targeting the heat shock protein (*groEL*) gene and the major surface protein4 gene (*msp4*), *A. capra* was detected in 129 (8.9%) of 1453 sheep and goat blood samples. The positive rate was higher in goats (9.4%, 89/943) than in sheep (7.8%, 40/510) (χ^2^ = 1.04, *p* > 0.05, *df* = 1). For sheep, *A. capra* was found in 17 sites from 2 provinces. The prevalence was 28.6% in sheep from Liaoning province, which was higher than in Henan Province (7.3%). For goats, *A. capra* was detected in 35 sites from 7 provinces. The prevalence varied from 0 to 19.4% in the goat sites examined. The prevalence rates were 19.4, 19.3, 10, 8.8, 6.8, 1.8, and 0% in goats from Guizhou province, Henan Province, Inner Mongolia Autonomous Region, Shanxi Province, Xinjiang Uygur Autonomous Region, Yunnan province, and Gansu province, respectively. Based on the analysis of the *A. capra* citrate synthase gene *(gltA*), two variants were identified. Variant I showed a high sequence similarity to the *A. capra*, which were previously reported in sheep, goats, *Ixodes persulcatus, Haemaphysalis longicornis, Haemaphysalis qinghaiensis*, and humans. Variant II was only found in Luoyang, Anyang, and Sanmengxia, of Henan province. To our knowledge, this is the first detection of this variant of *A. capra* in sheep and goat blood in China. Phylogenetic analysis based on *groEL* and *msp4* genes showed that the *Anaplasma* sp. sequences clustered independently from *A. capra* and other *Anaplasma* species with high bootstrap values. We found *A. capra* DNA in sheep and goats in China, providing evidence that sheep and goats can be infected by *A. capra*. We also found that this zoonotic pathogen is widely distributed in China. This study provides information for assessing the public health risks for human anaplasmosis.

## Introduction

*Anaplasma* species are zoonotic pathogens with tick vectors and mammalian reservoir hosts, and are common in tropical, subtropical, and some temperate regions of the world (Geurden et al., 2008; Tay et al., 2014; Yang et al., 2018). There are seven recognized species which are known to reside within the membrane enclosed vacuoles in the cytoplasm of blood and different cell types (Yang et al., 2015). The currently recognized species include *Anaplasma phagocytophilum, Anaplasma marginale, Anaplasma bovis, Anaplasma ovis, Anaplasma platys*, and *Anaplasma centrale. A. marginale* has been found in dairy cattle, and is the main inter-erythrocytic pathogen of bovine animals (Byaruhanga et al., 2018). *A. bovis* and *A. ovis* have been reported in both domestic and wild animals in various parts of the world (Liu et al., 2012; Lee et al., 2018). *A. platys* is the only classified rickettsial species known to infect platelets and cause infectious cyclic thrombocytopenia in dogs (Harvey et al., 1978; Ben Said et al., 2018; Vieira et al., 2018). *A. centrale* is characterized by progressive anemia associated with the presence of intra-erythrocytic inclusion bodies; it has also been used as a live vaccine against *A. marginale* for cattle in Australia, Africa, and South America (Rjeibi et al., 2017; Byaruhanga et al., 2018).

*Anaplasma phagocytophilum*, which was first recognized as a causative agent of human granulocytic anaplasmosis (HGA) in the USA in 1994, infects the neutrophils of humans and animals (Chen et al., 1994). *A. phagocytophilum* is now the most prevalent species of *Anaplasma* over broad areas where various tick species are vectors (Cao et al., 2000; Villar et al., 2015; Eisen, 2018). This species infects the neutrophils of cattle, sheep, goats, horses, dogs, hares, yaks, and 24 species of rodents (Kawahara et al., 2006; de la Fuente et al., 2016; Ochirkhuu et al., 2017; Rocchigiani et al., 2018). *A. phagocytophilum* has also been associated with HGA in China since 2008 (Wormser, 2016), and cases of HGA have been reported in Europe and North America (Thomas et al., 2009; Nichols Heitman et al., 2016).

*Anaplasma capra* was initially identified in 37 (31%) of 120 blood samples from asymptomatic goats in northern China in 2012, based on both the 16S rRNA gene (*rrs*) and the citrate synthase gene (*gltA*) (Li et al., 2015). The newly discovered *Anaplasma* species was then isolated from the samples during active surveillance of patients in a hospital in Heilongjiang Province, China; it was then identified in sheep and ticks in the northern and southern China (Li et al., 2015; Yang et al., 2016). The infection rates of ticks at the sites ranged from 0 to 78.6% (Yang et al., 2016, 2017, 2018). The disease caused by this pathogen is characterized by high hepatic aminotransferase concentrations, leucopenia, and thrombocytopenia. *A. capra* can be cultivated in human cell lines (HL-60, THP-1), but neither morulae nor other forms of this pathogen have been observed in the peripheral blood smears; only free bacteria or a few infected endothelial cells have been found (Li et al., 2015). Also, there is currently very little information about this pathogen, and identifying the reservoir host is essential to design disease control strategies.

To investigate the occurrence and prevalence of *A. capra* in sheep and goats in China, we collected blood samples from different regions in China and estimated the sheep and goats' reservoir capacity based on the infection prevalence. This information better defines the epidemiological role of sheep and goats.

## Materials and methods

### Sample collection

EDTA blood samples were collected from sheep and goats in rural areas from 52 sites in 8 provinces of China between March 2012 and October 2017. Guizhou and Yunan have a subtropical monsoon climate, with more rainfall in comparison to the temperate monsoon climate of Henan, Shanxi and Liaoning provinces. Xinjiang, Inner Mongolia, and Gansu have a temperate continental climate, and are dry with less rain. The sampling period was chosen to encompass the entire active season for ticks. Three to four specific flocks of sheep and goats were selected for sampling in each site. A total of 1,453 blood samples were collected in 10 ml sterile EDTA tubes.

### DNA extraction

DNA was extracted from 200 μl of EDTA blood samples individually using the Blood DNA Kit (OMEGA, Norcross, GA, USA) according to the manufacturer's instructions. The quantity and purity of the DNA were assessed by a NanoDrop spectrophotometer. Only those samples with at least 20 ng/μl of DNA were subjected to PCR assays. The extracted DNA was stored at −20°C until used.

### PCR assay

Nested PCR reactions targeting the *gltA* gene and conventional PCR targeting the heat shock protein (*groEL*) gene and the major surface protein4 gene (*msp4*) of *A. capra* were performed as previously described (Li et al., 2015; Yang et al., 2016). The primers and PCR conditions are described in Table 1. To prevent cross contamination, DNA extraction, amplification, and detection of PCR products were done in separate rooms. The PCR reactions were performed in an ABI 2720 thermal cycler (Life Technologies Holdings Pte Ltd., Singapore) with a total volume of 25 μl containing 2.5 μl of 10 × PCR buffer (Mg^2+^ Plus), 2.0 μl of each dNTPs at 2.5 mM, 1.25 U of Taq DNA polymerase (TaKaRa, Dalian, China), 2 μl of DNA for primary reactions or 2 μl of the primary PCR products for nested reactions, 0.5 μl of each primer (20 pmol), and 17.25 μl of distilled water. The PCR conditions comprised initial denaturation for 5 min at 94°C followed by 30 cycles of denaturation for 45 s at 94°C, annealing for 45 s at the temperatures listed in Table 1, and elongation for 45 s at 72°C.

**Table 1:** Primers and PCR amplification conditions for *A. capra*.

**Target gene**	**Primer name**	**Primer sequence (5′– 3′)**	**Annealing temperature (°C)**	**Amplicon size (bp)**	**References**
*gltA*	Outer-f	GCGATTTTAGAGTGYGGAGATTG	55	1,031	Yang et al., 2016
	Outer-r	TACAATACCGGAGTAAAAGTCAA			
	Inner-f	TCATCTCCTGTTGCACGGTGCCC	60	594	
	Inner-r	CTCTGAATGAACATGCCCACCCT			
*groEL*	Forward	TGAAGAGCATCAAACCCGAAG	55	874	Yang et al., 2017
	Reverse	CTGCTCGTGATGCTATCGG			
*msp4*	Forward	GGGTTCTGATATGGCATCTTC	53	656	
	Reverse	GGGAAATGTCCTTATAGGATTCG			

In each amplification, the DNA extracted from sheep infected with *A. capra* (GenBank accession nos. MG879297, MH174929, MH174932) was used as the positive control, and sterile water was used as the negative control.

### Sequencing of PCR products and phylogenetic analysis

The PCR products were visualized by UV transillumination in a 1.0% agarose gel following electrophoresis and staining with ethidium bromide. PCR amplicons were purified and sequenced on a 3730 DNA Sequencer (Applied Biosystems, Foster City, CA, USA), and analyzed by the BLASTN (http://www.ncbi.nlm.nih.gov/BLAST) and CLUSTALW (https://www.ebi.ac.uk/Tools/msa/clustalo/) programs. The nucleotide sequences were confirmed by bidirectional sequencing and also by sequencing a new PCR product if necessary.

Phylogenetic analysis was performed based on the sequence distance method using the neighbor-joining (NJ) algorithm using the MEGA 6.06 software. Confidence values for individual branches of the resulting tree were determined by bootstrap analysis with 1,000 replicates. The sequences obtained in this study were aligned with reference sequences downloaded from GenBank.

### Statistical analysis

Relationships between the aspects of the clinical history of each goat or sheep and the type of feeding model (grazing or household) were assessed using the Chi-square test with Yates' correction, and the differences were regarded significant when *p* ≤ 0.05.

### Accession numbers of nucleotide sequences

The representative sequences obtained in this study have been submitted and deposited in the GenBank database with accession numbers as follows: *gltA* genes (MG879297, MG879298, MG932656, MG932657), *groEL* genes (MH174929, MH174931), and *msp4* genes (MH174932, MH174933).

### Ethics statement

This study was conducted in accordance with the Chinese Laboratory Animal Administration Act (1988) after it was reviewed and its protocol was approved by the Research Ethics Committee of Henan Agricultural University. Appropriate permission was obtained from the farmer before the collection of blood specimens from the animals.

## Results

Of the 1,453 EDTA blood samples from sheep and goats, a total of 129 samples (8.9%) were *A. capra*-positive by the congruent results of three PCR amplification based on *gltA, groEL, msp4* locus. The positive rate was 7.8% (40/510) and 9.4% (89/943) in sheep and goats, respectively (Table 2). For sheep, *A. capra* was found in 17 sites from 2 provinces (Table 2). The prevalence rates were 28.6% in sheep from Liaoning province, and 7.3% in sheep from Henan Province (χ^2^ = 5.86, *df* = 1, 0.01 < *p* < 0.05). For goats, *A. capra* was detected in 35 sites from 7 provinces. The prevalence rate varied from 0 to 19.4% in the studied goats' sites, and the differences in positive rates were statistically significant (χ^2^ = 47.95, *df* = 6, *p* < 0.01) (Table 2).

**Table 2:** Prevalence distribution of *A. capra* isolates from sheep and goats from different provinces, China 2012–2017.

**Species**	**Geographic source**	**No. of sites**	**No. tested**	**No. positive (%)**	**Subtotal (%)**	***gltA* variant**
Sheep	Henan	15	496	36 (7.3)	40/510 (7.8)	Variant I, II
	Liaoning	2	14	4 (28.6)		Variant I
Goats	Henan	14	207	40 (19.3)	89/943 (9.4)	Variant I, II
	Shanxi	8	338	30 (8.8)		Variant I
	Inner Mongolia	2	40	4 (10)		Variant I
	Yunnan	4	218	4 (1.8)		Variant I
	Guizhou	2	36	7 (19.4)		Variant I
	Gansu	3	45	0 (0)		Variant I
	Xinjiang	2	59	4 (6.8)		Variant I
	Total	52	1,453	129 (8.9)		

**Figure 1:**
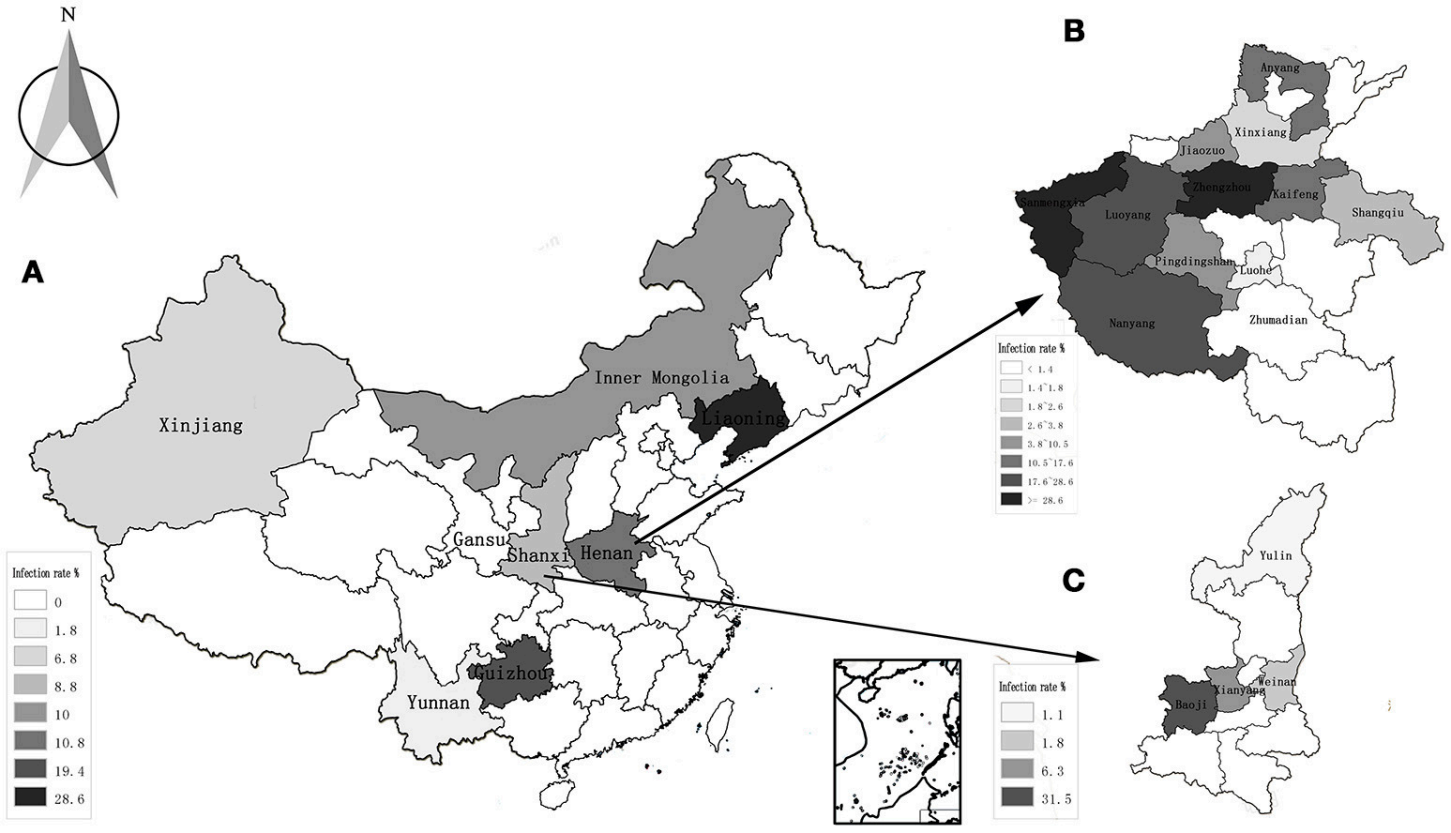
Distribution and infection of *A. capra* in all the studied regions. **(A)** The average rate of infection in 8 provinces of China; **(B)** the rate of infection in 12 countries of Henan province; **(C)** the rate of infection in 4 countries of Shanxi province.

The highest prevalence rates were 19.4% in goats from Guizhou province, while the lowest prevalence rates were 0% in the Gansu province (Table 2, Figure 1A). The highest prevalence rates were found in Zhengzhou (38.1%) of Henan province, Guiyang (35.3%) of Guizhou province, and Baoji (31.5%) of Shanxi Province, followed by Sanmenxia (28.6%), and Luoyang (24.9%) of Henan province (Figures 1B,C).

There were no statistically significant differences in the prevalence among the gender groups in sheep (χ^2^ = 1.33, *df* = 1, *p* > 0.05) and goats (χ^2^ = 0.28, *df* = 1, *p* > 0.05) (Table 3). However, there were some differences in prevalence amongst the age groups and feeding habits. Sheep that were <3 months old had the highest prevalence for both grazing and household feeding (66.7 and 12.5%, respectively), which may be related to a weakened immune system. The second highest prevalence was in sheep in the age group of 7 months − 1 year (32.4, 4.3%), while sheep in the age group of 3 months − 6 months (21.4, 0%) had the lowest prevalence. For goats, the highest prevalence was for the age group >1 year (14.5 and 4.4% for grazing and household feeding, respectively). The next highest was the age group of 3 months − 6 months (15.9, 1.4%), and the lowest prevalence was in the age group of <3 months (6.1, 0%) (Table 3). The prevalence was higher in grazing animals (15.5%) than household feeding animals (3.0%). The reason may be due to a greater exposure to ticks.

**Table 3:** Prevalence distribution of *A. capra* by age, gender, and feeding habits in sheep and goats from China 2012–2017.

**Age group**			**<3m (b/c d)**	**3–6m (b/c d)**	**7m−1y (b/c d)**	**>1y (b/c d)**	**Subtotal (b/c d)**
Sheep	Gender	Female	3/9 (33.3)	6/85 (7.1)	12/90 (13.3)	14/227 (6.2)	35/411 (8.5)
		Male	0/2 (0)	0/42 (0)	3/17(17.6)	2/38 (5.3)	5/99 (5.0)
	Feeding habits	Grazing	2/3 (66.7)	6/28 (21.4)	12/37(32.4)	6/22 (27.3)	26/90 (28.9)
		Household	1/8 (12.5)	0/99 (0)	3/70 (4.3)	10/243 (4.1)	14/420 (3.3)
Goats	Gender	Female	1/69 (1.4)	10/113 (8.8)	6/125 (4.8)	56/485 (11.5)	73/792 (9.2)
		Male	1/20 (5.0)	5/48 (10.4)	6/28 (21.4)	4/55 (7.3)	16/151 (10.6)
	Feeding habits	Grazing	2/33 (6.1)	14/88 (15.9)	12/112 (10.7)	52/359 (14.5)	80/592 (13.5)
		Household	0/56 (0)	1/73 (1.4)	0/41 (0)	8/181 (4.4)	9/351 (2.6)

*b/c, No. positive/No. tested, d: %*.

**Figure 2:**
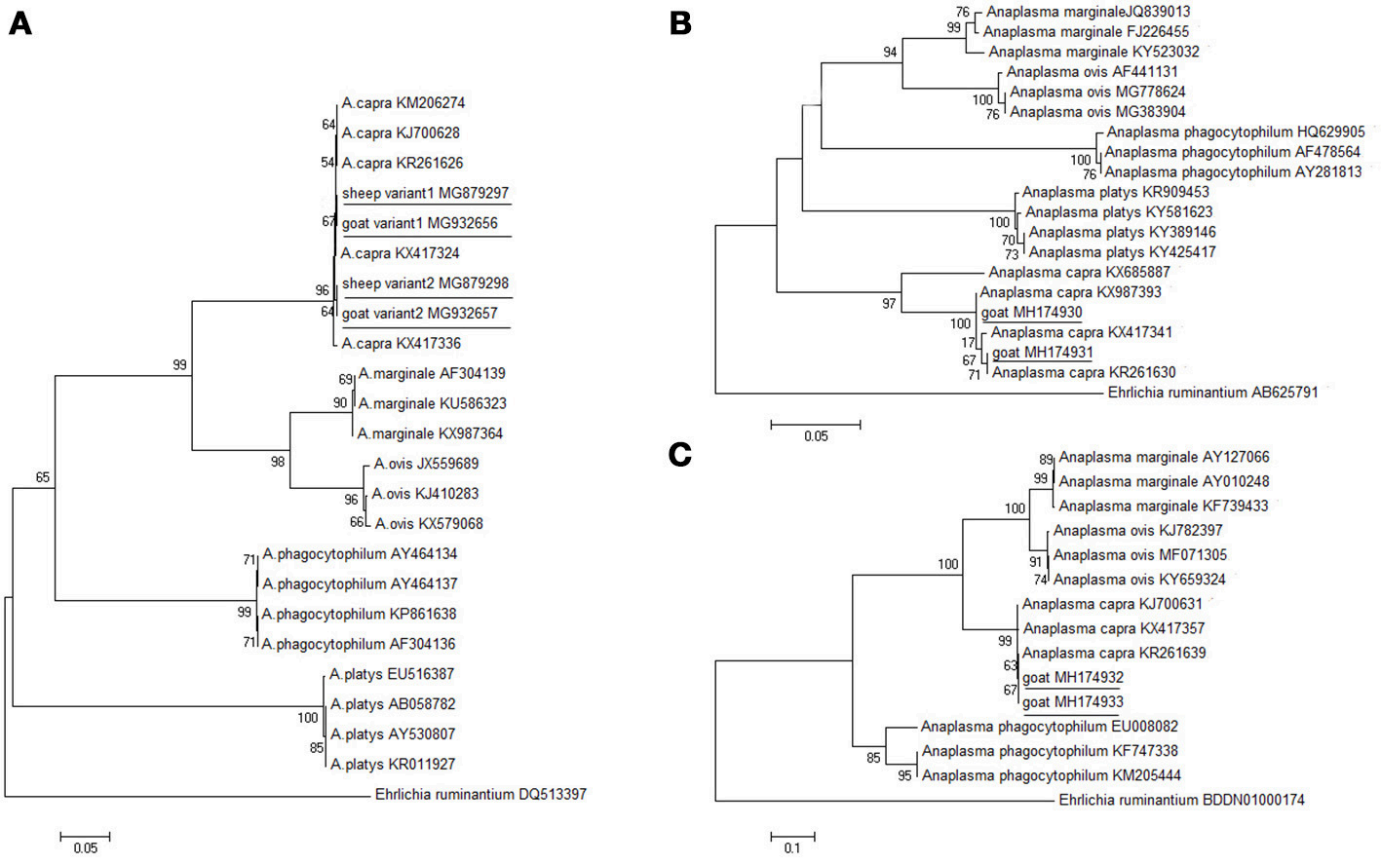
Phylogenetic analysis of *A. capra* identified in this study based on the *gltA* **(A)**, *groEL* **(B)**, and *msp4* **(C)** genes. The tree was constructed using the neighbor-joining method and the numbers on the tree indicate bootstrap values for the branch points. Numbers on the branches indicate percent support for each clade. *Ehrlichia ruminantium* was used as outgroup. The sequences from this study are underlined. The numbers of nucleotides were 594, 874, 656 bp in the final alignment of *gltA, groEL*, and *msp4*, respectively.

The molecular detection of *A. capra* isolates in sheep and goats was analyzed based on *gltA, groEL*, and *msp4* genes. The length (594 bp) of the *gltA* gene sequence from different sampling sites was used to determine the identity of the *Anaplasma* species. ClustalW analysis showed that most of the fragments of *A. capra* had similarities with the corresponding sequences detected in goats and humans (GenBank accession nos. KJ700628, KM206274). However, 20 out of 139 fragments (maximum of 99.3%) from Luoyang, Anyang, and Sanmenxia, in Henan province showed relatively little difference. The phylogenetic analysis of the *gltA* gene sequences demonstrated that the fragments identified in this study were in the same clade as members of *Anaplasma*, but distinct from other known *Anaplasma* species (Figure 2A). Further, the phylogenetic analysis revealed that the *A. capra* fragments identified in this study were in the same clade with the fragments reported earlier (Li et al., 2015; Yang et al., 2016).

Further analyses of *groEL* and *msp4* gene sequences showed 99.5% and 99.5% similarity between the *Anaplasma* sp. sequences (GenBank accession nos. KX987393, KR261639), respectively. The *groEL* sequences (GenBank accession nos. MH174929, MH174930, and MH174931) were 100, 100, and 100% identical to *A. capra* (GenBank accession no. KX987393). The *msp4* sequences (GenBank accession nos. MH174932, MH174933) were 100% and 100% identical to *A. capra* (GenBank accession no. KX417357). The phylogenetic analysis based on *groEL* and *msp4* genes showed that the *Anaplasma* sp. sequences clustered independently from *A. capra* and other *Anaplasma* species with high bootstrap values (Figures 2B,C), indicating potential novelty of the studied *Anaplasma* sp.

## Discussion

*Anaplasma* spp. are important tick-borne bacteria of veterinary importance that pose an increasing threat to the public and veterinary health (Fang et al., 2015). *A. phagocytophilum* and *A. capra* have been identified as pathogens of human anaplasmosis, from seven recognized *Anaplasma* species that infect various specific host cell types, such as erythrocytes, neutrophils, and platelets, depending on the host species (Troese et al., 2011; Kahlon et al., 2013; Tay et al., 2014; Baráková et al., 2017). *A. capra* was first recognized as asymptomatic in goats and was initially named by Li et al. (2015), who found *A. capra* in a human with a history of a tick bite in 2015 in Heilongjiang province, northern China (Li et al., 2015). Additional cases of human infection were identified from 622 febrile patients at the same site in 2010 (Li et al., 2012). The disease caused by *A. capra* was characterized by fever, headache, malaise, dizziness, and chills (Li et al., 2015). In another study, *A. capra* was inoculated into human cell lines (HL-60 and THP-1 cells) and morulae were observed at 24 days after inoculation, but no morulae or other blood agents were detected in any cells of peripheral blood smears on microscopic examination (Li et al., 2015).

Thus, microscopic detection of morulae in peripheral blood samples is unreliable for diagnosis of *A. capra* infection, unlike for *A. phagocytophilum* infection (Li et al., 2015). It has been proposed that *A. capra* might infect endothelial cells *in vivo*, similar to *A. phagocytophilum* and *A. marginale* (Munderloh et al., 2004). Further investigations are needed to better define *A. capra*'s host cells in human beings and infected vertebrates, such as sheep and goats.

We detected *A. capra* in blood from sheep and goats in China, regardless of feeding habits (whether grazing or fed in the household). The infection rate of *A. capra* was slightly higher in goats (9.4%, 89/943) than in sheep (7.8%, 40/510) as compared with a higher rate in sheep (16.3%) than in goats (12.3%) as previously reported (Yang et al., 2017). Further, the prevalence was higher for grazing animals (15.6%, 106/682) than household feeding animals (3.0%, 23/771) in the case of both sheep and goats. The difference in prevalence between the two feeding habits may be the result of exposure to ticks. There were no statistically significant differences in the prevalence between female (9.0%, 108/1203) and male (8.4%, 21/250) sheep or goats, in accordance with some previous studies (Yang et al., 2017). However, Belkahia et al. showed that, in dromedaries and sheep, the infection rates of *Anaplasma* spp. were significantly higher in females compared with males (Belkahia et al., 2015).

Our findings suggest that *A. capra* is widely distributed in China, and that sheep and goats can be infected by it. *A. capra* was also detected in *Ixodes persulcatus, Haemaphysalis longicornis*, and *Haemaphysalis qinghaiensis* in Heilongjiang province, Shandong Province, and Gannan Tibetan Autonomous Prefecture in Gansu, respectively (Sun et al., 2015; Yang et al., 2016). In addition, *A. capra* was detected in deer and free-living *Capricornis crispus* in Japan (Sato et al., 2009). Tick vector populations are expanding, and in turn increasing the risk of acquiring these *Anaplasma* species infections, thus this tick-borne pathogen is likely to be a growing concern for human and animal health (Chvostáč et al., 2018; Jaimes-Dueñez et al., 2018). To the best of our knowledge, to date, no other causative agent related to human anaplasmosis has been reported in China. Our study provides information for assessing the public health risks for human anaplasmosis.

Phylogenetic analysis of *A. capra* based on the *gltA, groEL*, and *msp4* genes demonstrated that the isolate sequences obtained from sheep created a separate clade within the genus *Anaplasma*, suggesting that these *A. capra* strains possess the same molecular characteristics. A novel *Anaplasma* species, closely related to *A. capra*, was identified from *H. qinghaiensis* in northwestern China (Yang et al., 2016). The *gltA* gene of that isolate exhibited the highest sequence similarity with *A. capra* (GenBank accession numbers KJ700628, KR261626, KM206274, KX417324), isolated from a goat, *H. longicornis, Homo sapiens*, and a sheep, respectively. This organism has also been detected previously in *Rhipicephalus microplus* (*Anaplasma* spp. strain WHBMXZ-125, GenBank accession no. KX987362) from Wuhan, China (Lu et al., 2017). The *Anaplasma* species that were identified in those domestic animals, ticks, and humans should be a single species according to the criteria for classification of bacteria.

These results indicate that there are at least three variants of *A. capra* circulating in nature. We found that one variant (GenBank accession nos. MG879297, MG932657) was in the same clade with the isolates from sheep, *H. qinghaiensis, H. longicornis*, and humans (GenBank accession nos. KX417308, KJ700628, KR261628, KX987362 and KM206274) in northwest China (Liu et al., 2012; Li et al., 2015; Lu et al., 2017; Yang et al., 2017). Another variant (GenBank accession nos. MG879298, MG932658) was identical to the following strains: LY63, SMX123, AY419, et al. To our knowledge, this is the first detection of this variant of *A. capra* in sheep and goats in Henan, China. The last sequence (GenBank accession no. KX417336) was isolated from goat blood in Guizhou, China (Yang et al., 2017). Further phylogenetic studies involving different gene markers are needed to better characterize the two *A. capra* variants observed in this investigation.

## Conclusions

This study was conducted to determine the occurrence of *A. capra* in blood samples of sheep and goats in China. The data showed that *A. capra* was widely distributed in China, especially in central China, and a high prevalence of grazing or household feeding sheep and goats can be infected by *A. capra*. Molecular characteristics suggest that this pathogen could be a substantial health threat to animals. Moreover, one novel variant of *gltA* gene which has never been reported before was found in this study. Further studies are needed to better understand the epidemiology and the pathogenicity of the *A. capra* circulating in China.

## Author contributions

YP and KW carried out the experiments, including PCR, cloning, sequencing, and data analysis. YP drafted the manuscript. SZ, YY, and JJ collected samples, while HW, FJ, LZ, and CN supervised the entire work. All authors read and approved the final version of the manuscript.

### Conflict of interest statement

The authors declare that the research was conducted in the absence of any commercial or financial relationships that could be construed as a potential conflict of interest.
